# Lipidomics Analysis of Multilamellar Bodies Produced by Amoeba *Acanthamoeba castellanii* in Co-Culture with *Klebsiella aerogenes*

**DOI:** 10.3390/pathogens12030411

**Published:** 2023-03-03

**Authors:** Magdalena Anna Karaś, Anna Turska-Szewczuk, Iwona Komaniecka, Barbara Łotocka

**Affiliations:** 1 Department of Genetics and Microbiology, Institute of Biological Science, Faculty of Biology and Biotechnology, Maria Curie-Skłodowska University, Akademicka 19, 20-033 Lublin, Poland; 2 Department of Botany, Faculty of Agriculture and Biology, Warsaw University of Life Sciences, Nowoursynowska 159, 02-776 Warsaw, Poland

**Keywords:** betaine lipids, phytoceramides, *Acanthamoeba castellanii*, multilamellar bodies, mass spectrometry, MS–MS, lipidomics

## Abstract

Multilamellar bodies (MLBs) are membrane-bound cytoplasmic organelles of lysosomal origin. In some protozoa, they were considered as lipid storage secretory organelles and feasible participants in cell-to-cell communication. However, for *Acanthamoeba castellanii*, similar vesicles were indicated only as possible transmission vectors of several pathogenic bacteria without attributing them biological roles and activities. Since amoebae belonging to the genus *Acanthamoeba* are not only of environmental but also of clinical significance, it is of great importance to fully understand their physiology. Thus, determination of MLB lipid composition could partly address these questions. Because MLBs are secreted by amoebae as a result of bacteria digestion, the co-culture technique with the use of “edible” *Klebsiella aerogenes* was used for their production. Lipids obtained from The MLB fraction, previously purified from bacterial debris, were analyzed by high-performance thin-layer chromatography, gas chromatography coupled with mass spectrometry, and high-resolution mass spectrometry. Lipidomic analysis revealed that in MLBs, a very abundant lipid class was a non-phosphorous, polar glycerolipids, diacylglyceryl-*O*-(N,N,N)-trimethylhomoserine (DGTS). Since DGTSs are regarded as a source of nitrogen and fatty acids, MLBs can be considered as lipid storage organelles produced in stress conditions. Further, the identification of phytoceramides and possible new betaine derivatives indicates that MLBs might have a distinct bioactive potential.

## 1. Introduction

Multilamellar bodies (MLBs) are round to oval cell organelles of lysosomal origin, which are formed by several concentric layers containing multiple thin electron-dense lamellae. MLBs have been found in various eucaryotic cell types, and depending on the location, their presence was associated with some medical disorders or normal physiological state. MLBs are also known to be produced during the phagocytosis process and secreted into the environment by different protozoa, including species of ciliates and free-living amoebae. For social amoeba *Dictyostelium discoideum*, it was indicated that the principal function of MLBs is storage and secretion of lipids. However, biochemical analyses of both lipids and proteins of *D. discoideum* MLBs also suggested their roles in cell-to-cell communication [1,2,3].

Betaine lipids (BLs) are a class of acylglycerolipids that have a quaternary amine alcohol ether linked to a diaclyglycerol moiety and are lacking in phosphorous. Three types of BLs have been reported to date—diacylgycerol-N,N,N-trimethylhomoserine (DGTS), diacylglycerylhydroxymethy-N,N,N-trimethylalanine (DGTA) and diacylglyceryl-N-carboxyhydroxymethylcholine (DGCC). Of these, DGTS is by far the most common in nature. BLs are widely distributed in lower plants and algae and can also occur in a limited number of species of non-photosynthetic microorganisms (bacteria, fungi, and protozoa) [4]. Some classes of DGTS were also present in a small amount in the cell membrane of *A. castellanii* [5]. Generally, isolated the plasma membrane of *Acanthamoeba* consists of approximately 27% lipids, 37% a polymer called lipophosphonoglycan (LPG), and 37% proteins [6]. Amoeba LPG belong to the class of glycoinositolphosphosphingolipids, which contains long-chain α-hydroxy fatty acids and as well as long-chain phytosphingosines [7].

DGTSs usually appear in cells in a large amount as a consequence of P-stress conditions, replacing typical phospholipids (PL) as phosphatidylcholine (PC), phosphatidylethanolamine (PE), and phosphatidylserine (PS) [4]. They are regarded as membrane structural components analogous to the phosphatidylcholine, as both have a zwitterionic nature, which they owe to the simultaneous presence of trimethylammonium and carboxyl groups. However, the physiological role of these lipids has not yet been fully determined. In *Nanochloropsis*, DGTSs were crucial for adapting to low temperatures and phosphate deficiency [8]; and in *Phaeodactylum tricornutum*, they served as a source of fatty acids for triglyceride formation in N-starved conditions [9]. However, the production of DGTS in plant fungal pathogen *Fusarium graminearum* promoted its virulence in phosphate-deficient apoplasts of maize [10]; and in the case of *Cryptococcus neoformans*, replacement of PC with DGTS contributed to virulence of fungi in an animal model [4]. It has also been shown that isolated MDGS and DGTS have anti-inflammatory activity, through downregulation of inducible nitric oxide synthase expression in LPS-stimulated RAW264.7 cells [11], thus indicating their immunomodulatory potential.

Free-living amoebae belonging to the genus *Acanthamoeba* are amphizoic and opportunistic protozoa that are ubiquitous in nature. Some strains can present a serious risk to human health as an agent of amoebae keratitis (AK) and life-threatening granulomatous amoebic encephalitis (GAE) [12]. As environmental predators, they feed on various bacteria; however, some of them that are mostly pathogenic are resistant to digestion. As a result of amoeba cytolytic activity, membranous vehicles filled with live bacteria can be expelled into the environment [13,14]. Since they are respirable, their size can serve as a public health risk as transmission vectors of pathogens but also as a virulence or immunomodulatory factor. In turn, the sequential assembly of specific lipids, dependent on the environmental conditions and the physiology of the relevant organism, plays a significant role in the function of biological membranes and a myriad of diseases. Thus, the aim of the present work was to obtain MLBs devoid of bacteria from *A. castellanii* and establish their lipid composition as an indirect indicator of their function. To the best of our knowledge, this is the first study concerning MLB production in *A. castellanii*.

## 2. Results

### 2.1. MLB Production in A. castellanii

Since, in earlier reports, it was shown that (i) MLBs are produced during the endocytolytic pathway by social amoeba Dictyostelium discoideum grazing on bacteria [1] and (ii) MLB-like vesicles containing live bacteria can be secreted by Acanthamoeba [13,14], it was necessary to obtain a fraction of such lamellar bodies free of bacteria cells and their debris prior to lipid extraction. Preparation of pure MLBs was carried out according to the procedure described by Paquet et al. [1], which consisted of feeding amoebae with *K. aerogenes* for the production of multilamellar bodies with subsequent centrifugation of MLBs secreted on sodium bromide density gradient. The one exception was omitting the stage of incubation of the primary obtained MLBs with a fresh batch of amoebae with increased phagocytic capacity. According to the authors, this step was necessary for elimination of bacterial contamination [1]. Unfortunately, our observations indicated that it significantly decreased the amount of previously acquired MLBs, being the source of lipids necessary for analysis.

**Figure 1:**
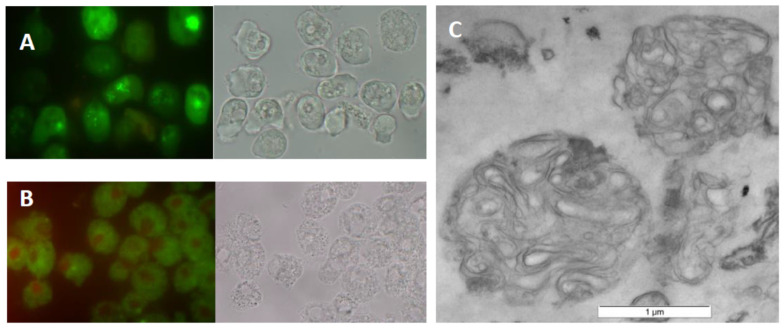
MLB production in *A. castellanii* in co-culture with *K. aerogenes*. (**A**) Metabolic active trophozoites of *A. castellanii* (green—stained with SYTO9) from monoxenic culture with bacteria. (**B**) Air-dried amoebae obtained from PYG medium as negative control (stained with both SYTO9 and PI). (**C**) TEM image of MLBs obtained from *A. castellanii*. LIVE/DEAD images collected on a Zeiss LSM780 laser scanning confocal microscope with excitation light using dual color channels. The same samples were also registered using transmitted light (left panels of (**A**,**B**)). The TEM image registered with FEI 268D ‘Morgagni’ transmission electron microscope (FEI Company, Hillsboro, OR, USA) equipped with an Olympus-SIS ‘Morada’ digital camera (Olympus). Scale bar 1 µm.

Since some microorganisms internalized by amoebae can lead to their death, we evaluated the vitality of *A. castellanii* by LIVE/DEAD assays during MLB production. The results obtained indicated that they were metabolically active until treated with starvation buffer (Figure 1A). In turn, to confirm MLB purity, their TEM images were registered. An overview indicated that MLBs were free of bacteria and are suitable for lipid extraction (Figure 1C). Additionally, to ensure that MLBs were formed in the presence of bacteria, TEM imaging of *A. castellanii* seeded on non-nutrient agar but without bacteria was also performed. The recorded images showed that the trophozoites, just before transformation into cysts, formed numerous vacuoles, probably filled with degraded cytoplasmic material, but without membrane lamellae inside (Supplementary materials Figure S1).

### 2.2. Chromatographic Analysis of MLB Components

Chloroform-methanol total extractable lipids (TLs) obtained from combined fractions of MLBs gave an average yield of 28.03% (5.25 mg) of their dry biomass (18.73 mg). The fatty acid composition of TLs subjected to mild alkaline hydrolysis and GC–MS analysis showed that C18:1 Δ^9^ (38.46 ± 0.87) was the most abundant FA, followed by C16:0 (21.43 ± 3.7%), C18:0 (20.75 ± 2.08%), C18:1 (4.4 ± 1.8%), C17:1 Δ^9^ (3.25 ± 0.7%), C20:4 Δ^5,8,11,14^ (2.41 ± 0.56%). The remaining ester-bound FAs, which had relative abundances of less than 1.5 mol%, are presented in Supplementary Table S1. Among them, no α-hydroxy fatty acids were identified.

**Figure 2:**
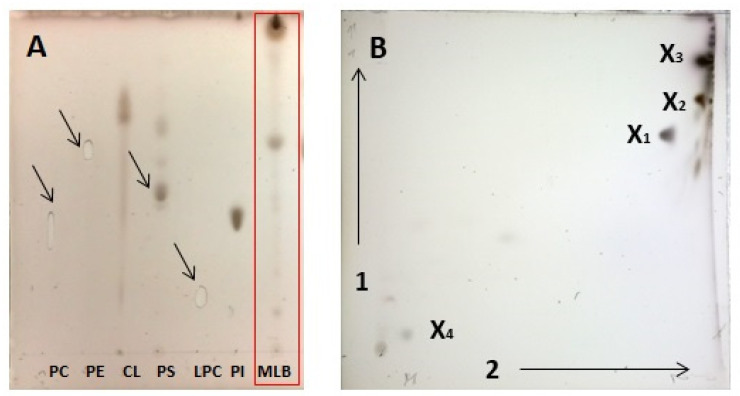
HPTLC profile of total lipids of MLBs produced by *A. castellanii*. (**A**). One-dimensional TLC of MLB lipids (red box) in comparison to commercial PL standards. TLC plates were developed in a *n*-propanol:propionic acid:chloroform:water (3:2:2:1, *v*/*v*/*v*/*v*) solvent mixture. (**B**). Two-dimensional TLC of MLB lipids. Used solvents: 1—first direction—chloroform/methanol/water (65:25:4, *v*/*v*/*v*/, 2—second direction—chloroform/methanol/acetic acid/water (90:15:10:3.5, *v*/*v*/*v*/*v*). Abbreviations used in spot assignments: PC, phosphatidylcholine; PE, phosphatidylethanolamine; CL, cardiolipin; PS, phosphatidylserine; LPC, lyso-phosphatidylcholine; PI, phosphatidylinositol; MLB, mixture of lipids derived from amoebae lamellar bodies; X1–X4, non-identified lipids; short arrows in (**A**) indicate the position of weak stained PLs spots.

The lipid profiles obtained by 1D and 2D HPTLC revealed only 3 and 4, respectively, pronounced spots after sulfuric acid charring (Figure 2). However, we could not assign them by comparison to migration of authentic phospholipid (PL) standards (Figure 2A and Supplementary Figure S3A); moreover, they did not stain with Molybdenum Blue reagent dedicated for phosphate-containing lipids. There were no such problems with identification of lipid classes derived from *K. aerogenes* whole cells (Supplementary Figure S3B). Thus, obtained data indicated that only non-phosphorous molecular species of lipids were present in amoebae MLBs.

### 2.3. Identification of Betaine Lipids in MLBs

In this situation, to achieve thorough identification of elemental composition, the total MLB lipid extract was analyzed by matrix-assisted laser desorption/ionization (MALDI–TOF) followed by direct-injection high-resolution tandem electrospray ionization–mass spectrometry (DI-ESI–MS) both in the positive and negative ion modes. Lipid species were identified based on the intact mass of the lipid and by MS^2^ fragmentation into structure-specific fragments that enabled compound classes and fatty acyl chains to be determined. In the positive ion mode, most lipids were recognized as protonated molecule [M + H]^+^ and one sodium adduct [M + Na]^+^ and the attribution yielded 14 polar lipid species distributed over betaine lipids monoacylglyceryl-*O*-(N,N,N)-trimethylhomoserine (MGTS), DGTS, and DGTA. Among three well-known classes of betaine lipids DGTS and DGTA are structural isomers. They are both characterized by the MS^2^ fragment (C_10_H_22_O_5_N)^+^ at *m/z* 236, MS^3^ fragments (C_7_H_14_O_2_N)^+^ at *m/z* 144 and (C_7_H_14_O_3_N)^+^ *m/z* 162, and neutral losses of fatty acyl chains as an acid (–RCOOH) and ketene (–R=C=O) derivatives in the MS^2^ spectrum. Additionally, neutral losses, 87 amu and sometimes 59 amu, can occur in their spectra. Therefore, distinguishing protonated ions of DGTS from DGTA is based on the intensity of two diagnostic precursor ions: [M + H]^+^ and the most intense in the MS^2^ spectrum. For DGTS, it is proposed that the protonated molecular ion was <20% of the intensity of the most intense fragment ion, while, for DGTA, [M + H]+ >> 50% of MS^2^ [15].

**Figure 3:**
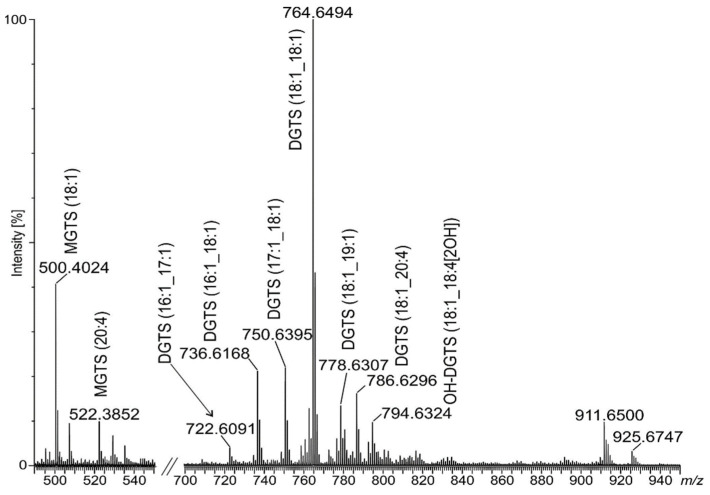
Spectrometric analysis of betaine lipids from MLBs of *A. castellenii*. Molecular ions registered as [M + H]^+^ adducts by ESI–MS in the *m/z* range 480–950. Region *m/z* 560–700 due to the presence of low-intensity unidentified ions was removed from the spectrum.

According to above rules, in our study, we identified two MGTS, nine DGTS, one oxidized DGTS (OH-DGTS), one DGTA molecular species, and one betaine lipid (DGTS or DGTA) without isomer attribution (Table 1, Figure 3). Peaks for DGTS at *m/z* 758, 764, 786, and DGTA *m/z* 772 are registered both in the DI-ESI–MS and MALDI–TOF spectra (Table 1 and Supplementary Figure S2).

**Figure 4:**
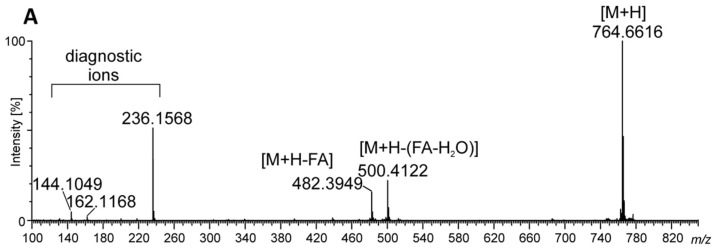

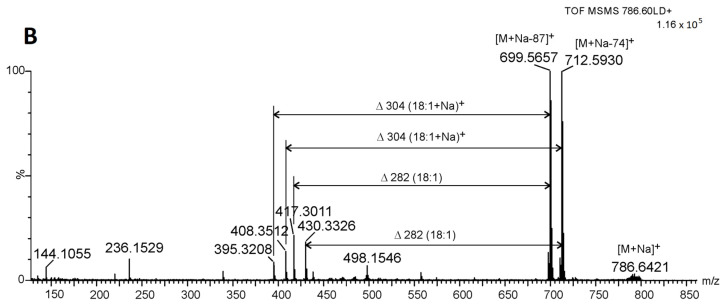
The MS^2^ spectrum and fragmentation patterns for DGTS (18:1/18:1) obtained from MLBs. (**A**) [M + H]^+^ ion in the DI-ESI–MS^2^ spectrum at *m/z* 764.6494. (**B**) [M + Na]^+^ ion in the MALDI–TOF–MS^2^ spectrum at *m/z* 786.6421 (Supplementary Figure S2).

The most abundant peak in the ESI–MS spectrum registered at *m/z* 764 represented DGTS (18:1/18:1) as a [M + H]+ ion (Figure 3 and Figure 4A), while, in the MALDI–TOF spectrum, the most abundant was at *m/z* 786, assigned as the [M + Na] adduct of the same lipid (Supplementary Figure S2). In the MS^2^ spectrum for this peak, neutral losses of 74 amu, 87 amu, losses of fatty acid and the presence of a diagnostic ion at *m/z* 236 were observed (Figure 4B). Such fragmentation was shown as characteristic for DGTS in the form of the [M + Na]^+^ adduct in spectrometric TOF analyses [16]. Among the identified DGTS molecular ions, one ion assigned as oxidized DGTS (18:1_18:4[2OH]) was also found. The analysis of its MS^2^ spectrum revealed two subsequent neutral losses of H_2_O and the product ions formed due to the loss of oxidized fatty acyl chains (acid and keto derivatives). Additionally, the product ion at *m/z* 236 was present in the MS^2^ spectrum; therefore, no product ions with oxidized polar head groups were observed (Table 1).

Identified betaine lipids were almost exclusively acylated with unsaturated fatty acids in both the *sn-1* and *sn-2* positions. On the basis of the loss of FA as neutral ions or as ketenes, we established that C16:1, C17:1, C18:1, C18:2, C18:4, C19:1 and C20:4 FA substituted the glycerol moiety (Table 1).

Among registered ESI–MS peaks in the positive mode, we could also observe two at *m/z* 911 and 925 (Figure 3) which we could not assign. However, their MS^2^ spectra were similar to fragmentation patterns of ions at *m/z* 772 and 786 assigned as DGTA (19:1_18:4) and DGTS (18:1_20:4), respectively, but with masses increased by 139 Da. This similarity reflected the occurrence of the MS^2^ ion at *m/z* 236, and some precursor ions created by neutral losses of fatty acids, as well as neutral loss of 87 amu. Moreover, neutral loss of 139 amu from [M + H]^+^ ions at *m/z* 911 and 925 formed precursor ions at *m/z* 772 and 786, respectively, in the MS^2^ spectra.

### 2.4. Ceramide Components in Acanthamoeba Derived MLBs

**Figure 5:**
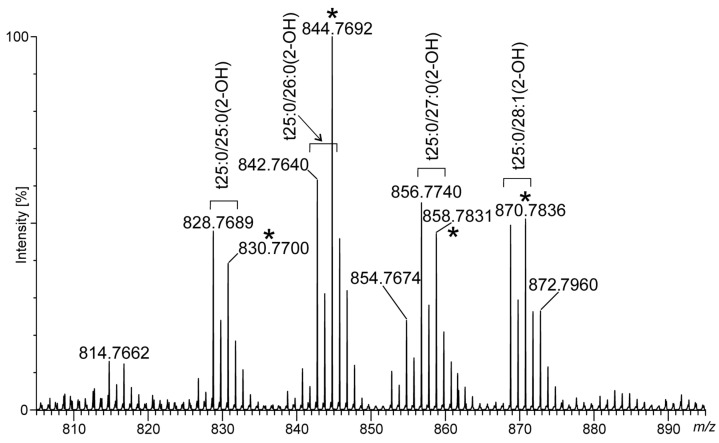
Phytoceramides identified in MLBs represented as pairs of isotopic ions [M + ^35/37^Cl]^−^ by ESI–MS in the negative ion mode registered in the region at *m/z* 810–900. In the spectrum, * is assigned to ^37^Cl adducts.

**Figure 6:**
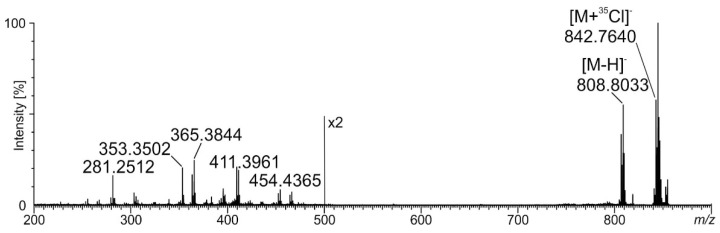
The MS^2^ spectrum for *t*25:0/26:0(2-OH) obtained from MLBs. Description of fragmentation included in text.

In the negative ion mode, we observed a series of peaks giving signals differing in mass by 14 Da (at *m/z* 828/830, 842/844, 856/858, and 868/870) (Figure 5), for which MS^2^ fragmentation revealed neutral losses of 34/36 amu typical for loss of ^35^Cl and ^37^Cl isotopes, respectively. Among these, the most pronounced peaks were at *m/z* 842/844, which, on the basis of the MS^2^ spectra, were assigned to the phytoceramid moiety *t*25:0/26:0(2-OH) recorded as [M + ^35/37^Cl]^−^ ions (Figure 6). The MS^2^ spectra revealed fragment ions which arose from the molecular ion at *m/z* 808 as a consequence of fragmentation processes activated by the hydroxyl group at C-4 of the phytosphingosine long-chain base (LCB), distinct phytoceramides from other ceramide families [17]. The cleavage of the C2-C3(OH) bond with charge residing on the fatty acyl end and LCB, respectively, gave rise to the N-acylethanolamine (NAE) anion at *m/z* 454 and the α-hydroxyaldehyde anion at *m/z* 353. The NAE anion after the rearrangement and loss of azetane (C_2_H_5_N) dissociated to an ion at *m/z* 411 (RCO_2_^−^ anion). The fragment ion related to *t*25:0 at *m/z* 365 arose from the molecular ion after neutral loss of 32 amu (HCHO + H_2_) followed cleavage of the C2-C3(OH) bond. Among the identified ions, those at *m/z* 365 and 353 are diagnostic ions for *t*25:0-LCB. They were also recognized in MS^2^ spectra for [M + ^35/37^Cl]^−^ ions at *m/z* 828/830, 856/858, and 868/870, corresponding to *t*25:0/25:0(2-OH), *t*25:0/27:0(2-OH), and *t*25:0/28:1(2-OH), respectively, on the basis of the fragmentation patterns. In their spectra, we recognized analogous ions as described above, and anions for α-hydroxy fatty acid moieties *m/z* 397, 425, and 437, respectively. Additionally, the formation of the ion (RCO_2_^−^ − [H_2_ + CO_2_]) in MS^2^ spectra, which is characteristic of the α-hydroxyl fatty acids, was differentiatiated from non-hydroxy-, β-hydroxy, or ω-hydroxy-fatty acid moieties [17].

### 2.5. Phospholipids Identified in MLBs by Spectrometric Analysis

Exhaustive searching of MS^2^ spectra for neutral losses and fragment ions characteristic of fatty acid-based lipids revealed only one class of PLs assigned as dimethyl-PE (14:0_16:1) registered as a scarce peak at *m/z* 688.5046 in the ESI–MS spectrum in the negative mode. Further, for a few other peaks, we identified a diagnostic PC fragment ion at *m/z* 184 (C_5_H_15_NO_4_P); however, the exact assignment to any PC molecular class was not possible because of the high error in mass accuracy. Additionally, despite the registration in the GC–MS chromatogram, with peaks related to 2,3-dihydroxypropyl hexadecanoate and 2,3-dihydroxypropyl octadecanoate suggesting the occurrence of ether type PLs liberated from MLBs, no assignment to this class was possible on the basis of the obtained MS^2^ spectra. Thus, data from spectrometric analysis showed that PLs were almost absent in MLBs. This was consistent with data from HPTLC chromatography.

## 3. Discussion

Extracellular vesicles of different structures and biological activities are released in distinct physiological and pathophysiological conditions by virtually every cell type. Generally, these vesicles are lipid bilayer-delimited particles. They represent a new and important topic, because they are a means of communication between cells, they can also be involved in removing cellular content and r participate in the immune response in higher organisms [18]. Among such vesicles are multilamellar bodies, which are lipid–protein complexes, creating concentric membrane layers. MLBs are found in numerous cell types, where they participate mainly in lipid storage and secretion [19].

Despite years of research on the physiology of *Acanthamoeba* and the pathogenesis of amoeba-related diseases, many aspects are still under investigation. In the case of AK, the eradication of the cysts is the main problem in treating the infection. However, an additional complication in diagnosis and treatment is caused by complex microbial AK infections accompanied by secondary infectious agents such as bacteria (also belonging to *Enterobacteriaceae*) or fungi [20,21]. These may exist as co-infectors or amoebae endosymbionts and contribute to: (i) the enhancement of binding of both *Acanthamoeba* and bacteria to the corneal endothelial cells, (ii) the enhancement of corneal toxicity in patients with AK, (iii) as well as the increased ability of amoebae to infect human hosts in the presence of the bacterial symbionts [21]. However, the degree to which bacterial endosymbionts actively affect the pathogenesis of AK and the mode of action presents contrasting results. Thus, new insight into these subjects is still necessary.

In this study, we demonstrated that *A. castellanii*, in the presence of ‘edible’ bacteria *K. aerogenes*, can produce bacteria-free MLBs, which are released into the environment in starved conditions. Additionally, we established that extractable lipids obtained from MLB particles consisted almost entirely of betaine lipids. Among them, different molecular species of MGTS, DGTS, and one DGTA were present (Table 1) with the predominant class assigned as DGTS (18:1/18:1) (Figure 3 and Supplementary Figure S2). The presence of DGTS as a minor component of the plasma membrane in *A. castellanii* trofozoites has already been reported [5]. According to Furlong [5], membranes of metabolically active amoebae were enriched mainly in DGTS substituted with *cis*-9-octadecanoic acid. Although no other classes of DGTS were registered in spectrometric analysis, the identification of minor amounts of hexadecenoic and octadecanoic acids might indicate a little more variety of this lipid class in trophozoites. Thus, it seems plausible that DGTS (18:1/18:1) in MLBs could appear as a result of remodeling of amoebae membranes, with bacteria endocytosis being a step in the MLB biogenesis [22]. Since, in our study, we identified 13 new molecular species of betaine lipids produced in *A. castellanii* MLBs, which were esterified almost entirely with unsaturated fatty acid residues of a different composition than previously established (also short PUFAs—18:4, 20:4, Supplementary Table S1), this indicates that their origin is distinct. According to some reports, when the content of DGTS in membranes increases, zwitterionic PLs such as PE and PC are almost completely simultaneously degraded. Thus, it can be concluded that phospholipids, mostly PC, provide diacylglycerol moieties for a part of DGTS synthesis [23,24]. Because eicosatetraenoic acid (C20:4 Δ^5,8,11,14^) is known to be mostly incorporated into PC and PE in *Acanthamoeba* [25], and in the course of the current study we established that few molecular species of DGTS are substituted with C20:4, it seems that such a remodeling system might also occur in MLBs. Additional evidence is the almost complete absence of PLs among MLB extractable lipids, which was confirmed by HPTLC chromatography and quadrupole spectrometry analysis. Based on overall fatty acid composition, DGTS is also substituted with C17:1 and C18:4, which were not identified in amoebae PLs [25]. Therefore, it can be concluded that membrane remodeling in the case of MLBs produced by *Acantahmoeba* is not simple substitution for PLs; however, the exact mechanism is as yet unknown. Similar conclusions were drawn for DGTS biosynthesis in different species of microalgae [26]. Among identified betaine lipids, one oxidized OH-DGTS occurred. It is not clear if it arose as a result of auto-oxidation or was naturally present in MLBs. The existence of oxidized subclass of DGTS among polar lipids was confirmed for microalgae *Chlorococcum amblystomatis* [27].

We describe here for the first time the occurrence of DGTA in *Acanthamoeba* membranes. In addition to DGTS and DGTA being structural isomers, it was shown that DGTS serves as precursor for DGTA biosynthesis. In *Ochromonas danica*, the biosynthesis of DGTA from DGTS involved decarboxylation and recarboxylation of the polar part and simultaneous deacylation and reacylation of the glycerol moiety [28]. If the synthesis of DGTA in *Acanthamoeba* followed the same pattern, this could have explained the lack of DGTS esterified with the same FA residues as in the case of DGTA in total lipid preparation from MLBs.

Generally, membrane lipid remodeling contributes to acclimation to environmental stress conditions. During phosphate deprivation, some organisms relocate Pi from membrane lipids to other metabolic pathways. As a result, the content of PLs decreases what is complemented with the increase in BLs [8]. According to our findings, *A. castellanii* co-cultured with ‘edible’ bacteria on non-nutrient medium produced MLBs enriched in betaine lipids. Thus, it can be assumed that the depletion of nutrients in the environment stimulated that process, as well the replacement of PLs by BLs in membranes, which reflected Pi turnover in cells. Because, hypothetically, DGTS may constitute a pool of FA for the production of triacylglycerols, as in the case of the microalgae *Phaeodactylum tricornutum*, MLBs can be considered as recycling machinery of different kinds of storage lipids, sequentially arising in response to environmental conditions [9]. An additional proof for this may be data obtained by Denoncourt et al. [29], who showed that MLBs isolated from *D. discoideum* were subsequently internalized and efficiently digested by another pool of this social amoeba, and also by ciliates of the genus *Tetrahymena*. We made similar observations for *A. castellanii* in the course of this study (data not presented), resulting in a change in the protocol for obtaining MLBs compared to the original [1], as mentioned in the Materials and Methods section.

MLBs containing BLs liberated into surroundings could perform different biological activities. In order to survive in the host cells, *Acanthamoeba*, as with other pathogens, might use strategies to bypass immune responses, for example through lowering inflammatory reactions. In the course of amoeba keratitis, macrophages act as a first line of defense and eliminate significant numbers of *Acanthamoeba* spp. [30]. Since betaine lipids as DGTS/DGTA are not produced by the mammals, they can have immunogenic properties. According to some reports, purified MGTS and DGTS were able to inhibit NO production in RAW264.7 macrophages by downregulating the expression of nitric oxide synthase; similarly, providing evidence, BLs can be considered as potential anti-inflammatory agents [11,31]. Thus, in the AK course, especially with complex-type AKs with co-infection with bacteria from *Enterobacteriaceae*, amoebae might use this strategy to release abundant MLBs in BLs or re-build cell membranes, substituting PLs into betaine lipids. Moreover, BLs being polar lipids and replacing PLs may affect membrane organization and associated virulence factors through lipid–lipid and lipid–protein interactions, thus influencing the course of pathogenesis. However, these hypotheses will require further research.

We also reported here that MLBs are enriched in phytoceramides, in which elemental composition consists of LCB and FAs, similar to in the LPG of *Acathamoeba.* Phytoceramides are common in plants, filamentous fungi, yeasts, and lower animals; however, those with very long LCB were found in human skin cells [7]. Since it is known that sphingolipids play a crucial role in cell signaling, microbial pathogenesis, etc., in defense against oxidative stress, which allow colonization in the host [32,33], MLBs may also play an important role in the pathogenesis of diseases caused by *Acathamoeba* spp.

Spectrometric analysis revealed the occurrence two unusual ions in the ESI–MS spectra, with masses increased by 139 Da in comparison to some molecular species of DGTS, and their fragmentation patterns were similar to betaine lipids. This might indicate that they are a new kind of DGTS derivative or that they are unidentified adducts. However, determining this was beyond the scope of this work.

As mentioned earlier in this manuscript, lipid composition is an important characteristic from the viewpoint of membrane properties: fluidity, flexibility, and selective permeability. Lipid composition can also determine membrane vesicle properties including lamellarity and the efflux rate of inside-enclosed compounds [34,35]. Therefore, the results of research as ours can also be used in liposome production technology. Knowledge about naturally occurring multilayer bodies and the composition of their lipids, which determines the organization of internal membranes, may be useful in the future in the design of multilayer liposomes that are carriers of, e.g., drugs.

## 4. Materials and Methods

### 4.1. Materials

PLs used as standards for HPTLC, MALDI–TOF, DI-ESI–MS and MS–MS analysis (1,2-dipalmitoyl-*sn*-glycero-3-phosphocholine, 2-dipalmitoyl-*sn*-glycero-3-phosphoethanolamine, and cardiolipin sodium salt from bovine heart) were from Sigma-Aldrich Chemical Co., St. Louis, MO, USA or (1-palmitoyl-2-hydroxy-*sn*-glycero-3-phosphocholine, 1-palmitoyl-2-oleoyl-*sn*-glycero-3-phosphoinositol ammonium salt, and 1-palmitoyl-2-linoleoyl-*sn*-glycero-3-phospho-L-serine sodium salt) from Avanti Polar Lipids, Alabaster, MA, USA. Chemicals for MS systems (chloroform, methanol, 2-propanol, and acetonitrile were all of LC–MS grade) and Molybdenum Blue reagent for HPTLC were obtained from Merck (Darmstadt, Germany), and 2,5-dihydroxybenzoic acid (DHB) and trifluoroacetic acid (TFA) were obtained from Sigma-Aldrich (St. Louis, MO, USA). Viability Kit LIVE/DEAD^TM^ BacLight^TM^ from Invitrogen was used in the viability assay.

### 4.2. Strains and Culture Conditions

The Neff strain *Acanthamoeba castellanii* (ATCC 30010) was grown in 300 mL conical flasks in a PYG (Peptone–Yeast–Glucose) medium with shaking as described previously [14]. A Bürker chamber was used to estimate the density of amoebae in the culture. *K. aerogenes* bacteria were stored on LB agar; and prior to conducting co-culture with amoebae, they were transferred into tubes with liquid LB medium and grown on a rotary shaker (180 rev/min) at 37 °C for 24 h. To produce secreted MLBs, *A. castellanii* was grown in the presence of *K. aerogenes* at an MOI of 1: 5000 on 15 cm NNA agar plates. Co-cultures were run at room temperature until the complete disappearance of the bacterial lawn (approximately 3–5 days). The progress of the process was monitored using a phase-contrast microscope. Prior to co-culture, both the amoebae and bacteria were triple washed with PBS; and before applying onto plates, they were mixed in appropriate proportions.

### 4.3. Purification of MLBs

When the amoebae were almost entirely consumed by bacteria, 6 mL of starvation buffer (2 mM Na_2_HPO_4_, 14.7 mM KH_2_PO_4_, 100 mM sorbitol, 100 μM CaCl_2_, and 1% PYG) [1] was poured into each plate and then co-cultures were detached from the medium with sterile scrapers. The purification of MLBs was performed according to Paquet et al. [1], with some modifications. Briefly, the collected co-cultures suspended in buffer were transferred to Falcon tubes and centrifuged to settle *A. castellanii* cells (450× *g* for 5 min at 4 °C). Then, the supernatant was centrifuged at 4000× *g* for 10 min at 4 °C, and the obtained pellet with MLBs suspended in PBS was intensively vortexed to release bacterial residues from membrane formations. Finally, centrifugation was repeated under the same conditions. To separate MLBs from other vesicles and residual free bacteria, the pellet was suspended in PBS followed by the application of 1 mL aliquots on the top of glass tubes containing a 6 mL sodium bromide density gradient (1 mL of six different densities ranging from 1.0 to 1.5 g/mL). The tubes were centrifuged at 3220× *g* for 45 min at room temperature. The clearly visible yellowish aggregates between layers from particular tubes were collected and washed two times with PBS and centrifuged at 17,000× *g* for 10 min. The purified MLBs were kept at 4 °C. The purity of the preparations was confirmed by transmission electron microscopy (TEM). As the negative control of MLB production, axenic cultures of amoebae on NNA agar plates were used.

### 4.4. LIVE/DEAD Assay

To determine the viability of amoebae co-cultured with *K. aerogenes* for MLB production, we employed LIVE/DEAD staining. Amoebae samples were taken for analysis at the end of the culture and before starvation buffer was applied. Images were collected on a Zeiss LSM780 laser scanning confocal microscope using 488 nm excitation light from an Argon laser for SYTO9 (a vital dye that penetrates the plasma membrane) and 561 nm excitation light from a DPSS laser for propidium iodide (PI)—a nuclear dye that does not penetrate the plasma membrane. Metabolically active cells can be identified by green fluorescence due to Syto 9 but not red with PI, and dead cells were labelled with both SYTO9 and PI. As a negative control, air-dried *A. castellanii* cells from PYG medium were used. Drying results in membrane damage and affects the penetration of both dyes.

### 4.5. TEM 

The preparations for an electron microscope were made as before [14]. Both MLBs and 48 h trophozoites from axenic cultures on the NNA agar plates were harvested and prefixed for 2 h at 4 °C in 2.5% glutaraldehyde in 0.1 M cacodylate buffer (pH 7.3) with 3% of sucrose and 2.5 mM CaCl_2_. The samples were then washed twice in 0.1 M cacodylate buffer, post-fixed in 1% osmium tetroxide for 1 h at 4 °C, stained with 1% aqueous uranyl acetate, and dehydrated with an ascending ethanol series. The resulting pellets were then infiltrated with and embedded in LR White resin. After polymerization (50 °C for 2 days), the resin blocks were sectioned, post-stained with uranyl acetate and lead citrate, and examined using an FEI 268D ‘Morgagni’ transmission electron microscope (FEI Company, Hillsboro, OR, USA) equipped with an Olympus-SIS ‘Morada’ digital camera (Olympus).

### 4.6. Lipid Extraction

Before extraction, MLBs were triple washed with deionized water and lyophilized, while *K. aerogenes* bacteria were washed once with an aqua solution of 0.5 M NaCl and triple washed with deionized water, and finally also lyophilized. Total lipids were extracted from both lyophilized MLBs and bacteria using the Bligh and Dyer protocol with ultrasonification in aquatic environments. Since lipid recoveries *are* affected by the *extraction* method, the second extraction was performed in acidic conditions (0.5% 6 M HCl) to avoid losses of acidic phospholipids. Lipid extraction was performed three times and the combined organic fractions were dried on a rotary evaporator. The obtained samples were next suspended in a chloroform:methanol (2:1, *v*/*v*) mixture, transferred onto a Pasteur pipette filled with a plug of filter paper (Whatman GF/C), and collected into new tubes, which were then stored at −20 °C until further analysis.

### 4.7. Lipid Separation by TLC

Total lipids obtained from MLBs suspended in chloroform:methanol (2:1) and commercial PLs standards spotted onto HPTLC silica gel 60 plates (Merck, Darmstadt, Germany) and applied to one-dimensional TLC (1D-TLC) and two-dimensional TLC (2D-TLC). For 1D-TLC, a solvent *n*-propanol:propionic acid:chloroform:water (3:2:2:1, *v*/*v*/*v*/*v*) mixture was used. For 2D-TLC, chloroform:methanol:water (65:25:4) and chloroform:methanol:acetic acid:water (90:15:10:3,5) mixtures were used for the first and second dimensions, respectively [36]. For the visualization of the lipids, plates were sprayed with 10% sulfuric acid in 50% methanol and charred at 160 °C or with iodine vapor. Phosphorus was detected using phosphomolybdate reagent.

### 4.8. Gas Liquid Chromatography with Mass Spectrometry (GC–MS) Analysis of Fatty Acids

For fatty acid analysis, samples of total lipids derived from MLBs and bacteria were subjected to saponification in 0.8 M NaOH in methanol (80 °C, 1 h). Then, samples were acidified to pH 2.0 with 6 M HCl and evaporated to dryness with a vacuum evaporator. Extraction was carried out with chloroform:water (1:2, *v/v*); and after phase separation, the organic layer containing liberated FAs, prior rinsing with water, was dried on an anhydrous sodium sulfate packed column and the chloroform was removed in a nitrogen stream. Obtained FAs were converted into methyl esters by methanolysis (1 M HCl/MeOH; 85 °C, 1.5 h) and re-extracted prior to GC–MS analysis carried out on an Agilent Technologies 7890A (Agilent Technologies, Wilmington, DE, USA) gas chromatograph connected to a 5975C MSD (inert XL EI/CI, Agilent Technologies, Wilmington, DE, USA) detector, equipped with an HP-5MS capillary column (Agilent Technologies, 30 m × 0.25 mm). Hel was used as a carrier gas with a flow rate of 1 mL/min. The temperature gradient was as follows: 150 °C (5 min) to 310 °C at 5 °C min^−1^, kept for 10 min. For the analysis of hydroxyl fatty acids, samples containing FA methyl esters were trimethylsilylated with HMDS/TMCS/pyridine (3:1:9, *v*/*v*/*v*) (Sigma-Aldrich, St. Louis, MO, USA) prior to injection. The results are presented as ± S.D. of three independent measurements.

### 4.9. Electrospray Ionization–Mass Spectrometry (ESI–MS) Analysis of Lipids

ESI–MS spectrometry was performed with a SYNAPT G2-S*i* HDMS instrument (Waters Corporation, Milford, MA, USA) operating in the positive ion electrospray mode. Acquisition of the data was performed at a range of 100–1600 *m/z*, using MassLynx software version 4.1 SCN916 (Waters Corporation, Wilmslow, UK). Mass spectrometer conditions for both the positive and negative ion modes were as follows: capillary voltage 1.00 kV, sampling cone 30 V, and source offset 80 V. Ion source temperature was established at 120 °C and desolvation temperature was 500 °C. Cone gas flow was set at 100 L/h and desolvation gas flow was 800 L/h. For MS^2^ experiments, isolated precursor ions were fragmented using increasing the collision voltage from 15 V to 90 V. Data were collected for 120 s for each precursor ion. Mass spectra were assigned with a multipoint external calibration using sodium iodide (Sigma-Aldrich, St. Louis, MO, USA). Samples for analysis were dissolved in chloroform/methanol (2:1, *v*/*v*) at a concentration of 10 µg/µL. A 200 µL sample was transferred to the 2 mL glass vial and diluted with 1.8 mL of a 2-propanol/acetonitrile/water (2:1:1, *v*/*v*/*v*) mixture. Then, the sample was filtered using a PTFE syringe filter (0.22 µm, Alfatech Technology) and transferred to the new glass vial. Samples were injected by infusion, at a flow rate of 10 µL/min, and MS spectra were registered by 5 min. MS data were acquired in the positive ion mode for monoacylglyceryl 3-O-4′-(N,N,N-trimethyl) homoserine (MGTS), diacylglyceryl 3-O-4′-(N,N,N-trimethyl) homoserine (DGTS), and diacylglycerylhydroxymethy-N,N,N-trimethylalanine (DGTA) identified as [M + H]^+^ ions. The MS spectrum was acquired in the negative ion mode for phosphatidylethanolamine (PE), phosphatidylglycerol (PG), phosphatidylserine (PS) identified as [M − H]^−^ ions, and phytoceramides identified as [M + ^35/37^Cl]^−^ ions. Where the fatty acid moieties were identified but their stereochemistry is unknown, an underscore (_) is used between the two fatty acid substituents. If substitution of a fatty acid at *sn*-1 and *sn*-2 is known, a solid is used between the fatty acid substituents [15]. The results presented are from three independent measurements.

### 4.10. MALDI–TOF–MS and MS–MS Spectrometry

For MALDI–TOF mass spectrometry analysis, a 0.5 M 2,5-dihydroxybenzoic acid (DHB) solution in methanol containing 0.1% trifluoroacetic acid (TFA) was used as matrix. The extract of MLB total lipids was diluted in chloroform/methanol (2:1, *v*/*v*) to a concentration of 5 μg/μL and directly applied onto the target plate as 1 μL droplets, followed by the addition of 1 μL DHB matrix solution. After drying and crystallization at room temperature, samples were analyzed by MALDI–TOF–MS and MS–MS.

All MALDI–TOF mass spectra were acquired on a Waters SYNAPT G2-Si HDMS instrument (Waters Corporation, Milford, MA, USA) equipped with a 1 kHz Nd:YAG laser system. Acquisition of the data was performed using MassLynx software version 4.1 SCN916 (Waters Corporation, Wilmslow, United Kingdom). Spectra were recorded in the positive ion polarities. For MS^2^ experiments, isolated precursor ions were fragmented using a collision voltage of 50–60 V. Data were collected for 120 s for each ion separately. Mass spectra were assigned with multipoint external calibration using red phosphorous (Sigma-Aldrich, St. Louis, MO, USA).

## 5. Conclusions

In this study, we performed lipidomics analysis of MLBs produced by *Acanthamoeba* in the presence of *K. aerogenes* to address their physiological role and possible biological activities, especially in complex microbial AK, in future research. Currently, due to a high abundance of ether-linked glycerolipids containing a betaine moiety, we propose that MLBs are mostly the recycling machinery of storage lipids. However, MLBs may have a wider variety of functions because they contain phytoceramides.

## Figures and Tables

**Table 1:** Betaine lipids identified in the total lipids of MLBs derived from *A. castellanii* by DI–ESI–MS and MALDI–TOF in three independent analyses. Presented fragmentation patterns obtained by DI–ESI–MS^2^ analysis.

Lipid Ion/Formula	Obs *m*/*z*	Calc *m*/*z*	Product Ion
MGTS (18:1_0) + H C_28_H_54_O_6_N	500.4024	500.3951	(C_7_H_14_O_2_N) 144.1049:MS3, (C_7_H_16_O_3_N) 162.1168:MS3, (C_10_H_22_O_5_N) 236.1568:MS2
MGTS (20:4_0) + H C_30_H_52_O_6_N	522.3852	522.3795	(C_7_H_14_O_2_N) 144.1049:MS3, (C_7_H_16_O_3_N) 162.1168:MS3, (C_10_H_22_O_5_N) 236.1568:MS2, NL(C_2_H_4_N(CH_3_)_3_) 435.3200:MS2
*^a^* DGTS/DGTA (32:0) + H C_42_H_82_O_7_N	712.6046	712.6091	-
DGTS (16:1_17:1) + H C_43_H_80_O_7_N	722.6091	722.5935	(C_7_H_14_O_2_N) 144.1049:MS3, (C_7_H_16_O_3_N) 162.1168:MS3, (C_10_H_22_O_5_N) 236.1568:MS2, NL[FA(17:1)] 454.3679:MS2, NL[FA(17:1)] + H_2_O (472.3710):MS2, NL[FA(16:1)] 468.3758:MS2, NL[FA(16:1)] + H_2_O (486.3916):MS2
DGTS (16:1_18:1) + H C_44_H_82_O_7_N	736.6168	736.6091	(C_7_H_14_O_2_N) 144.1049:MS3, (C_7_H_16_O_3_N) 162.1168:MS3, (C_10_H_22_O_5_N) 236.1568:MS2, NL[FA(18:1)] 454.3679:MS2, NL[FA(18:1)] + H_2_O (472.3710):MS2, NL[FA(16:1)] 482.3949:MS2, NL[FA(16:1)] + H_2_O (500.4024):MS2
DGTS (17:1_18:4) + H C_45_H_78_O_7_N	744.5764	744.5778	(C_7_H_14_O_2_N) 144.1020:MS3, (C_7_H_16_O_3_N) 162.1134:MS3, (C_10_H_22_O_5_N) 236.1521: MS2, NL[FA(18:4)] 468.3679:MS2, NL[FA(18:4)] + H_2_O (486.3679):MS2,NL(C_2_H_4_N(CH_3_)_3_) 657.5064:MS2
DGTS (17:1_18:1) + H C_45_H_84_O_7_N	750.6395	750.6248	(C_7_H_14_O_2_N) 144.1049:MS3, (C_7_H_16_O_3_N) 162.1168:MS3, (C_10_H_22_O_5_N)^+^ 236.1568: MS2,NL[FA(18:1)] 468.3758:MS2, NL[FA(17:1)] 482.3949:MS2, NL[FA(18:1)] + H_2_O (486.3916):MS2, NL[FA(17:1)] + H_2_O (500.4024):MS2
*^b^* DGTS (18:1_18:4) + H C_46_H_80_O_7_N	758.6021	758.5935	(C_7_H_14_O_2_N) 144.1020:MS3, (C_7_H_16_O_3_N) 162.1134:MS3, (C_10_H_22_O_5_N) 236.1521: MS2, NL[FA(18:4)] 482.3869:MS2, NL[FA(18:4)] + H_2_O (500.3942):MS2, NL(C_2_H_4_N(CH_3_)_3_) 671.5272:MS2
*^b^* DGTS (18:1/18:1) + H C_46_H_86_O_7_N	764.6494	764.6404	(C_7_H_14_O_2_N) 144.1049:MS3, (C_7_H_16_O_3_N) 162.1168:MS3, (C_10_H_22_O_5_N) 236.1568:MS2, NL[FA(18:1)] 482.3949:MS2, NL[FA(18:1)] + H_2_O (500.4024):MS2
DGTS (18:1_19:1) + H C_47_H_88_O_7_N	778.6428	778.6561	(C_7_H_14_O_2_N) 144.1049:MS3, (C_7_H_16_O_3_N) 162.1168:MS3, (C_10_H_22_O_5_N) 236.1568:MS2, NL[FA(19:1)] 482.3949:MS2, NL[FA(18:1)] 496.3694:MS2, NL[FA(19:1)] + H_2_O (500.4024):MS2, NL[FA(18:1)] + H_2_O (514.3845):MS2
DGTS (18:2_20:4) + H C_48_H_82_O_7_N	784.6108	784.6091	(C_7_H_14_O_2_N) 144.1020:MS2, (C_10_H_22_O_5_N) 236.1521: MS2, NL[FA(18:2)] 504.3665:MS2, NL[FA(18:2)] + H_2_O (522.3866):MS2, NL[C_2_H_4_N(CH_3_)_3_] 697.5432:MS2
*^b^* DGTS (18:1_20:4) + H C_48_H_84_O_7_N	786.6296	786.6248	(C_7_H_14_O_2_N) 144.1049:MS3, (C_7_H_16_O_3_N) 162.1168:MS3, (C_10_H_22_O_5_N) 236.1568:MS2, NL[FA(20:4)] 482.3949:MS2, NL[FA(20:4)] + H_2_O (500.4122):MS2, NL[C_2_H_4_N(CH_3_)_3_] 699.5732:MS2
OH-DGTS (18:1_18:4[2OH]) + H C_46_H_84_O_9_N	794.6324	794.6146	(C_7_H_14_O_2_N) 144.1049:MS3, (C_7_H_16_O_3_N) 162.1168:MS3, (C_10_H_22_O_5_N) 236.1568:MS2, NL[FA(18:4)[2OH]] 482.3949:MS2, NL[FA(18:4)[2OH]] + H_2_O (500.4122):MS2, NL[FA(18:1)] 512.3668:MS2, NL[FA(18:1)] + H_2_O (528.3685):MS2, NL(2H_2_O) 758.6138, NL(H_2_O) 776.6221
*^b^* DGTA (19:1_18:4) + H C_47_H_82_O_7_N	772.6129	772.6091	(C_7_H_14_O_2_N) 144.1020:MS3, (C_7_H_16_O_3_N) 162.1134:MS3, (C_10_H_22_O_5_N) 236.1521:MS2, NL[FA(18:4)] 496.3694:MS2, NL[FA(18:4)] + H_2_O (514.3760):MS2, NL[C_2_H_4_N(CH_3_)_3_] 685.5473:MS2

*a*—molecular species identified exclusively on the basis of the exact mass registered by MALDI–TOF; *b*—structures additionally confirmed by MALDI–TOF–MS^2^.

## Data Availability

The data presented in this study are available in the article and Supplementary Materials.

## Supplementary Materials

The following supporting information can be downloaded at: https://www.mdpi.com/article/10.3390/pathogens12030411/s1, Figure S1: Representative TEM image of *A. castellanii* trophozoit cultured axenically for 48 h on NNA agar plates; Table S1: Classes and content (given in mol%) of ester-bound fatty acid residues identified in lipids extracted from MLBs using the Bligh and Dyer protocol. Figure S2: Spectrometric analysis of betaine lipids from MLBs of *A. castellenii*. Figure S3: The 2D HPTLC chromatograms of (A) commercial standards and (B) phospholipids extracted from whole cells of *Klebsiella aerogenes*. Table S2: Lipids identified in whole cells of *K. pneumoniae* by DI-ESI–MS. For some molecular species, fatty acids were established by MALDI–TOF–MS–MS fragmentation. Figure S4: The MS^2^ mass spectrum and fragmentation pattern for a selected ion corresponding to phospholipid PE (16:1/17:1) [M − H]^−^ obtained from total lipid extracts from whole cells of *K. aerogenes.*
