# Supplementary material for: Lipidomics Analysis of Multilamellar Bodies Produced by Amoeba Acanthamoeba castellanii in Co-Culture with Klebsiella aerogenes
Source: Pathogens. 2023 Mar 3;12(3):411. doi: 10.3390/pathogens12030411 (PMC10057378; doi:10.3390/pathogens12030411)
Supplement: Supplementary file 1 [file pathogens-12-00411-s001.zip › Supplementary materials.pdf]

Lipidomics Analysis of Multilamellar Bodies Produced by Amoeba *Acanthamoeba castellanii* in Co-Culture with *Klebsiella aerogenes*

Magdalena Anna Karaś [1,*], Anna Turska-Szewczuk [1], Iwona Komaniecka [1] and Barbara Łotocka [2]

[1] Department of Genetics and Microbiology, Institute of Biological Science, Faculty of Biology
and Biotechnology, Maria Curie-Skłodowska University, Akademicka 19, 20-033 Lublin, Poland
[2] Department of Botany, Faculty of Agriculture and Biology, Warsaw University of Life Sciences,
Nowoursynowska 159, 02-776 Warsaw, Poland
* Correspondence: magdalena.karas@mail.umcs.pl

Supplementary Materials

1. The MLBs production inside Acanthamoeba castellanii

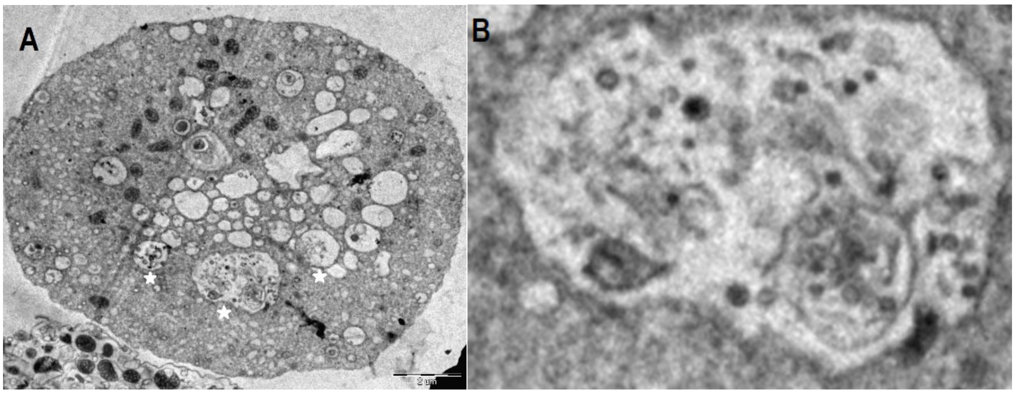

Figure S1. Representative TEM image of *A. castellanii* trophozoit cultured axenically for 48 hours on NNA agar plates. A. and **B.** magnified image of an autophagic-like vacuole; The white stars denote autophagic-like vesicles containing completely or partially digested mitochondria or cytosolic compartments.

2. Lipidomic analysis of MLBs derived of Acanthamoeba castellanii

Table S1. Classes and content (given in mol %) of ester bound fatty acids residues identified in lipids extracted from MLBs using the Bligh and Dyer protocol.

Fatty acid	mol %
14:0	1.055 ± 0.18
15:0	*tr*
16:1$^{\Delta 7}$	1.87 ± 0.72
**16:0**	**21.43 ± 3.7**
17:1*	3.25 ± 0.7
17:0	*tr*
18:3*	*tr*
18:2$^{\Delta 9,12}$	1.36 ± 0.47
**18:1$^{\Delta 9}$**	**38.46 ± 0.87**
18:1*	4.4 ± 1.8
**18:0**	**20.75 ± 2.08**
20:4$^{\Delta 5,8,11,14}$	2.41 ± 0.56
20:3$^{\Delta 8,11,14}$	1.15 ± 0.11

20:2$^{\Delta 11,14}$	1.4 ± 0.07
20:0	*tr*

Data were determined by GC-MS analysis of the FAs methyl esters subsequent to saponification (0.8 M NaOH/50 % MeOH; 1 h/ 80°C). The results are presented as ± S.D. of three independent measurements. *

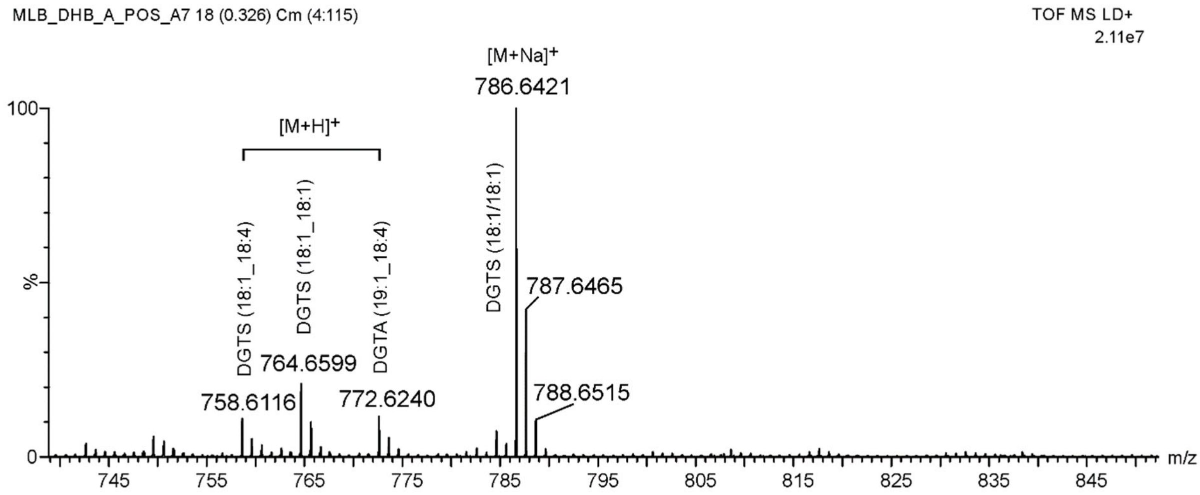

Figure S2. Spectrometric analysis of betaine lipids from MLBs of *A. castellenii*. Ions registered as $[M + H]^+$ and $[M + Na]^+$ adducts by MALDI-TOF in *m/z* range 700 - 850.

3. *Lipids derived of whole cells Klebsiella aerogenes*

3.1. *HPTLC analysis*

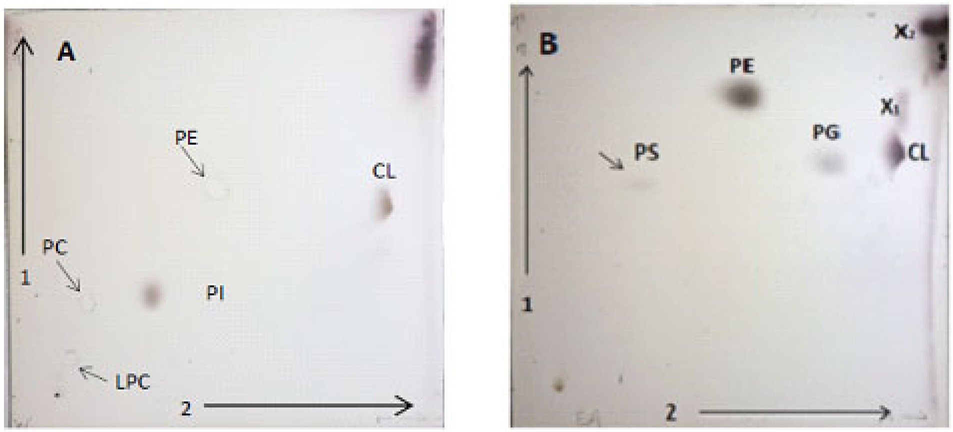

Figure S3. 2-D HPTLC chromatograms of **A**) commercial standards **B**) phospholipids extracted from whole cells of *Klebsiella aerogenes*. Abbreviations: PC - phosphatidylcholine, PE - phosphatidylethanolamine, CL - cardiolipin, PS - phosphatidylserine, LPC - lysophosphatidylcholine, PI - phosphatidylinositol; Used solvents: 1 – first direction - chloroform/methanol/water (14:6:1, v/v/v), 2- second direction chloroform/methanol/acetic acid (13:5:2) [15].

3.2. *Fatty acids identified in total lipids of K. aerogenes*

Among fatty acids liberated by saponification (conditions as for data in Table 1S) from total lipids of *K. aerogenes* and derivatizated to methyl esters identified by GC-MS: C16:0 (41.97 ± 0.6 mol%), *cyc*17:0 (29.53 ± 0.19 mol%), and *cyc*19:0 (14.92 ± 1.31 mol%), C18:1$^{\Delta 9}$ (5.14 ± 0.09 mol%), C14:0 (3.9 ± 0.55 mol%). In trace amount (< 2.0 %) also registered: C15:0, C16:1, C17:0, C18:0. The results are presented as ± S.D. of three independent measurements.

3.3. *DI-ESI-MS and MALDI-TOF MS² analysis of lipids K. aerogenes*

Table S2. Lipids identified in whole cells *K. pneumoniae* by DI-ESI-MS. For some molecular species fatty acids were established by MALDI-TOF MS² fragmentation. Abbreviations for PLs are given in Figure 3S. *c* - cyclopropane ring structure.

Lipid (acyl carbons: double bonds)	Adduct	Formula	Observed *m/z*	Calculated *m/z*
**PE (30:0)**	$[M + H]^+$	$C_{35}H_{71}NO_8P$	664.4926	664.4917
	$[M + Na]^+$	$C_{35}H_{70}NO_8PNa$	686.4781	686.4737
	$[M - H]^-$	$C_{35}H_{69}NO_8P$	662.4811	662.4761
**PE (31:1)**	$[M + H]^+$	$C_{36}H_{71}NO_8P$	676.4960	676.4917
	$[M + Na]^+$	$C_{35}H_{70}NO_8PNa$	698.4815	698.4737
	$[M - H]^-$	$C_{36}H_{69}NO_8P$	674.4771	674.4761
**PE (32:1)**	$[M + H]^+$	$C_{37}H_{73}NO_8P$	690.5097	690.5074
	$[M + Na]^+$	$C_{37}H_{72}NO_8PNa$	712.4986	712.4893
	$[M - H]^-$	$C_{37}H_{71}NO_8P$	688.4937	688.4917
**PE (32:0)**	$[M + H]^+$	$C_{37}H_{75}NO_8P$	692.5300	692.5230
	$[M + Na]^+$	$C_{37}H_{74}NO_8PNa$	714.5040	714.5050
	$[M - H]^-$	$C_{37}H_{73}NO_8P$	690.5127	690.5074
**PE (16:0/*c*17:0)**	$[M + H]^+$	$C_{38}H_{75}NO_8P$	704.5281	704.5230
	$[M + Na]^+$	$C_{38}H_{74}NO_8PNa$	726.5137	726.5050
	$[M - H]^-$	$C_{38}H_{73}NO_8P$	702.5155	702.5074
**PE (34:1)**	$[M + H]^+$	$C_{39}H_{77}NO_8P$	718.5469	718.5387
	$[M + Na]^+$	$C_{39}H_{76}NO_8PNa$	740.5228	740.5206
	$[M - H]^-$	$C_{39}H_{75}NO_8P$	716.5226	716.5230
**PE (35:1)**	$[M + H]^+$	$C_{40}H_{79}NO_8P$	732.5620	732.5543
	$[M + Na]^+$	$C_{40}H_{78}NO_8PNa$	754.5458	754.5363
	$[M - H]^-$	$C_{40}H_{77}NO_8P$	730.5462	730.5387
**PE (*c*17:0_*c*19:0)**	$[M + H]^+$	$C_{41}H_{79}NO_8P$	744.5551	744.5538
	$[M + Na]^+$	$C_{41}H_{78}NO_8PNa$	766.5355	766.5397
**PG (*c*17:0_16:0)** **PG (*c*19:0_14:0)**	$[M - H]^-$	$C_{39}H_{77}NO_{10}P$	733.5012	733.5020
**PG (35:1)**	$[M - H]^-$	$C_{41}H_{78}NO_{10}P$	788.5470	788.5442
**PS (36:1)**	$[M - H]^-$	$C_{42}H_{79}NO_{10}P$	788.5470	788.5442

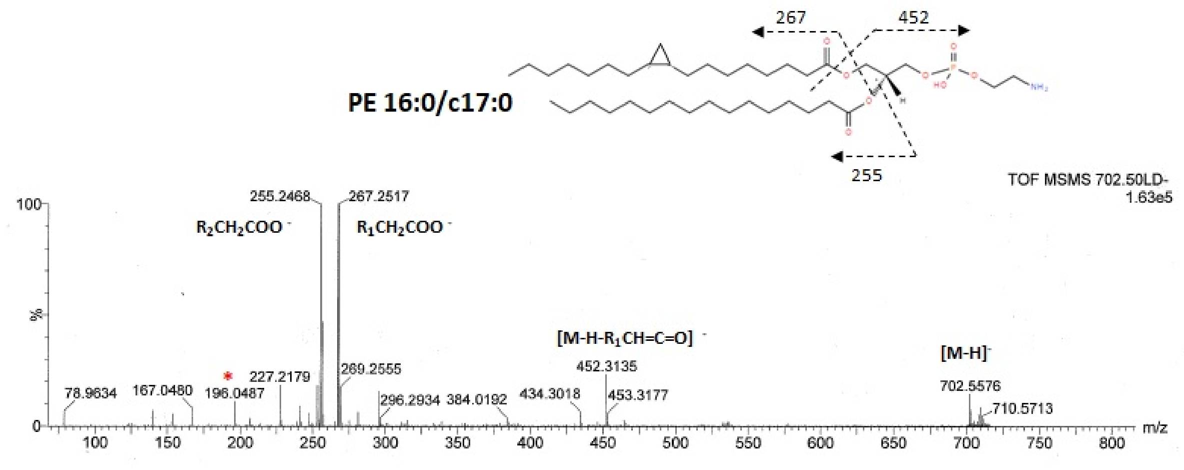

Figure S4. The MS^2 mass spectrum and fragmentation pattern for a selected ion corresponding to phospholipid PE (16:0/c17:0) $[M – H]^-$ obtained from total lipid extract from whole cells of *K. aerogenes.*
