# Supplementary figures and images for: Lipidomics Analysis of Multilamellar Bodies Produced by Amoeba Acanthamoeba castellanii in Co-Culture with Klebsiella aerogenes
Source: Pathogens. 2023 Mar 3;12(3):411. doi: 10.3390/pathogens12030411 (PMC10057378; doi:10.3390/pathogens12030411)

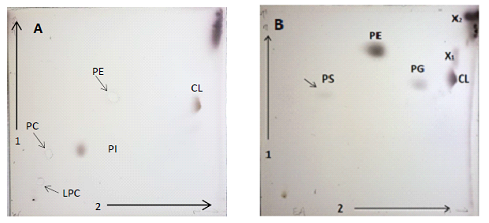

Supplement: Supplementary file 1 [file pathogens-12-00411-s001.zip › Figure S3.tif]

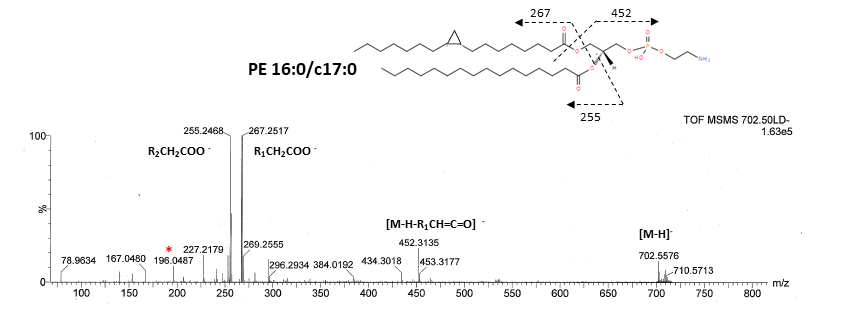

Supplement: Supplementary file 1 [file pathogens-12-00411-s001.zip › Figure S4.tif]

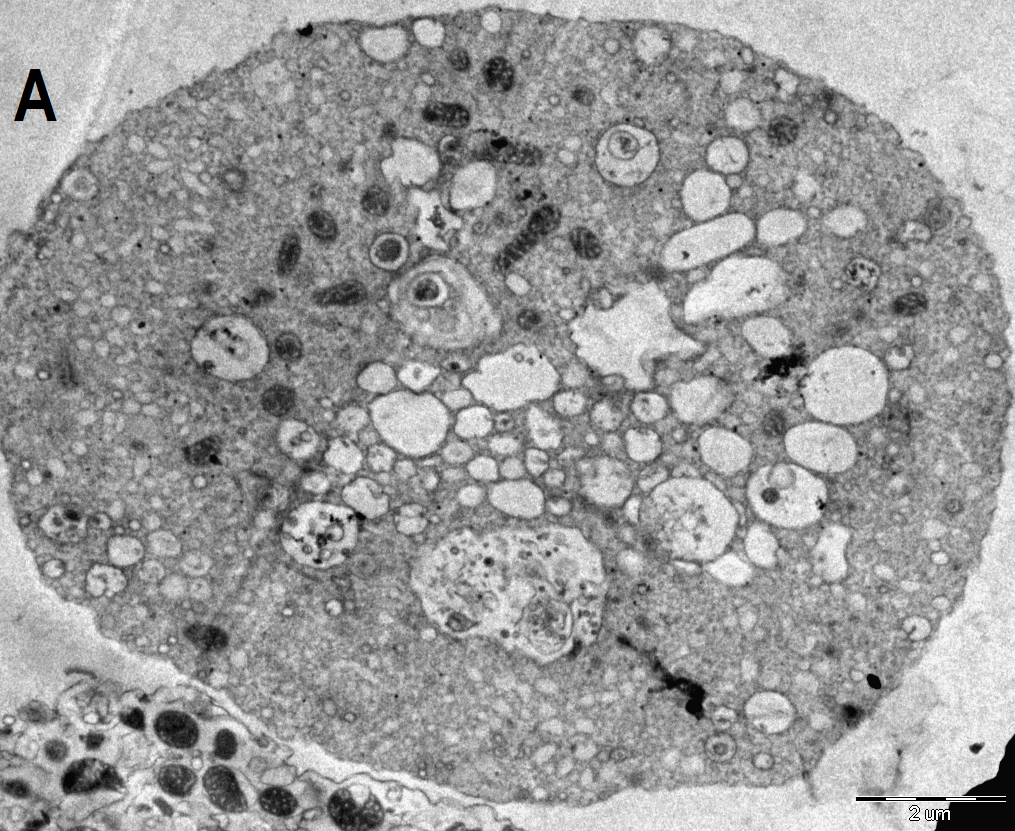

Supplement: Supplementary file 1 [file pathogens-12-00411-s001.zip › Figure S1A.tif]

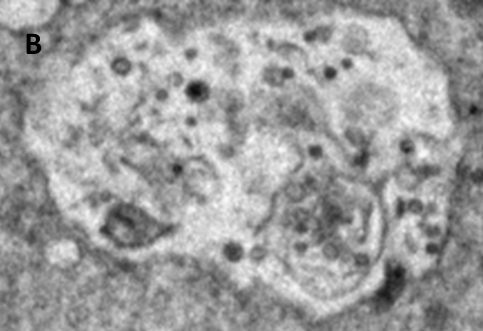

Supplement: Supplementary file 1 [file pathogens-12-00411-s001.zip › Figure S1B.tif]

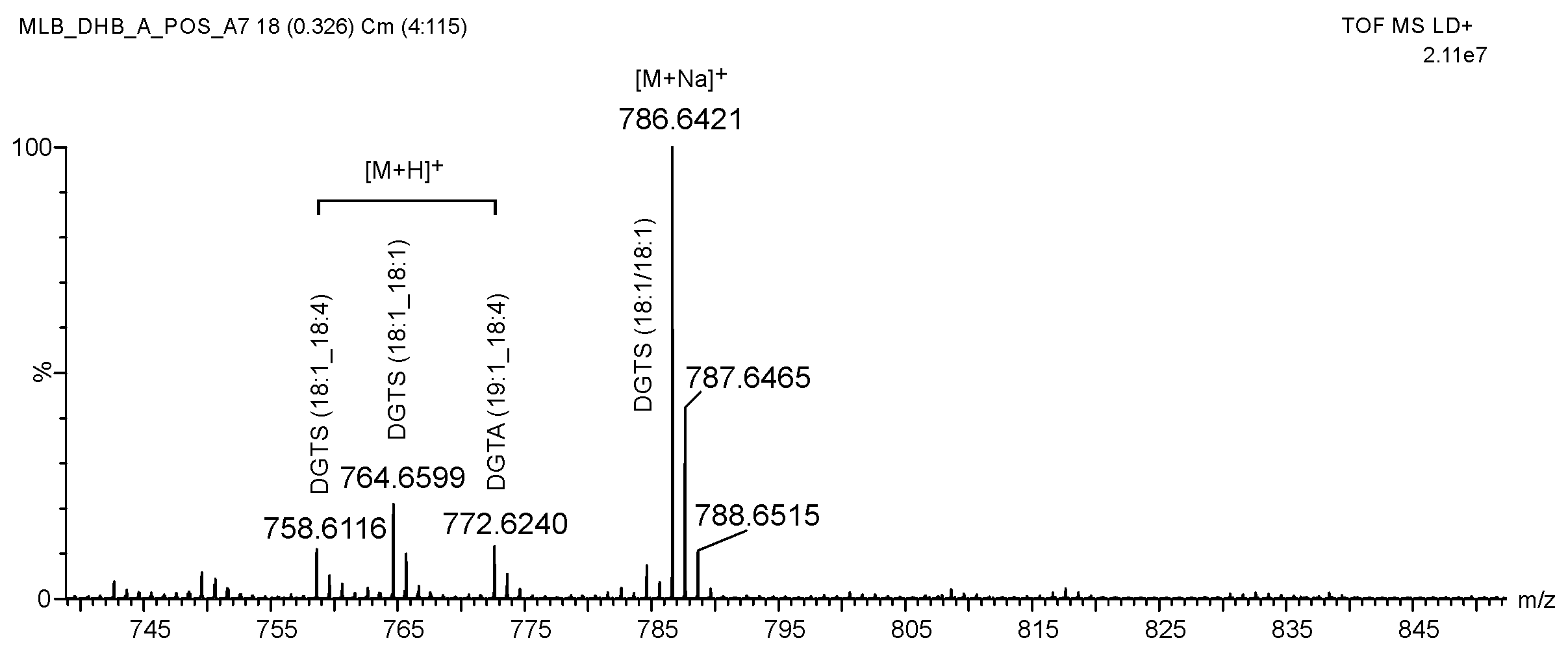

Supplement: Supplementary file 1 [file pathogens-12-00411-s001.zip › Figure S2.tif]
